# Chemically Modified Zein- and Poly(methyl vinyl ether-co-maleic anhydride)-Based Core–Shell Sub-Micro/Nanoparticles for Essential Oil Delivery: Antibacterial Activity, Cytotoxicity, and Life Cycle Assessment

**DOI:** 10.3390/nano16020139

**Published:** 2026-01-20

**Authors:** Liudmyla Gryshchuk, Kyriaki Marina Lyra, Zili Sideratou, Fotios K. Katsaros, Sergiy Grishchuk, Nataliia Hudzenko, Milena Násner, José Gallego, Léo Staccioli

**Affiliations:** 1 PS Resins GmbH, Lembergerstr. 94, 66955 Pirmasens, Germany; 2 Leibniz-Institut für Verbundwerkstoffe GmbH, Erwin-Schrödinger-Str. 58, 67663 Kaiserslautern, Germany; nataliia.hudzenko@ivw.uni-kl.de; 3 Institute of Nanoscience and Nanotechnology, National Centre of Scientific Research “Demokritos”, Aghia Paraskevi, 15310 Attiki, Greece; k.lyra@inn.demokritos.gr (K.M.L.); z.sideratou@inn.demokritos.gr (Z.S.); f.katsaros@inn.demokritos.gr (F.K.K.); 4 Department of Applied Logistics and Polymer Sciences, Campus Pirmasens, Kaiserslautern University of Applied Sciences, Carl-Schurz-Str. 10-16, 66953 Pirmasens, Germany; sergiy.grishchuk@hs-kl.de; 5 ARDITEC Association, 48 Avenue Mont-Rabeau, 06200 Nice, France; milena.nasner@arditec.net (M.N.); jose.gallego@arditec.net (J.G.); leo.staccioli@arditec.net (L.S.)

**Keywords:** nanocapsules, bio-based polymers, essential oils, antimicrobial, toxicity, life cycle assessment

## Abstract

The threat of antimicrobial resistance (AMR) and the need for sustainable disinfectants have spurred interest in natural antimicrobials such as essential oils (EOs). However, their application is limited by volatility, poor water solubility, and cytotoxicity. Herein, we present the development of bio-based core–shell sub-micro-/nanocapsules (NCs) with encapsulated oregano (OO), thyme (TO), eucalyptus (EuO), and tea tree (TTO) oils to enhance antimicrobial (AM) performance and reduce cytotoxicity. NCs were synthesized via a nanoencapsulation method using chemically modified zein or poly(methyl vinyl ether-co-maleic anhydride) (GZA) as shell polymers, with selected EOs encapsulated in their core (encapsulation efficacy > 98%). Chemical modification of zein with vanillin (VA) and GZA with either dodecyl amine (DDA) or 3-(glycidyloxypropyl)trimethoxysilane (EPTMS) resulted in improvement in particle size distributions, polydispersity indices (PDIs) of synthesized NCs, and in the stability of the NC-dispersions in water. Antibacterial testing against *Staphylococcus aureus* and cytotoxicity assays showed that encapsulation significantly reduced toxicity while preserving their antibacterial activity. Among the formulations, GZA-based NCs modified with EPTMS provided the best balance between safety and efficacy. Despite this, life cycle assessment revealed that zein-based NCs were more environmentally sustainable due to lower energy use and material impact. Overall, the approach offers a promising strategy for developing sustainable, effective, and safe EO-based antibacterial agents for AM applications.

## 1. Introduction

Throughout human history, infectious diseases have had significant influence on societies [1]. With the invention of antibiotics and the introduction in many countries of mandatory vaccination, it was possible to fight a number of diseases, or to restrain their global spread [2]. Unfortunately, antimicrobial resistance (AMR) and insufficient levels of vaccination, especially in low-income countries, international travel, population migration, and the emergence of pathogens, infectious diseases represent a constant and increasing threat to human health and well-being. It is important to note that many infectious diseases, such as diarrhea, pneumonia and acute respiratory infections, healthcare-associated infections, helminths and parasitic infections, etc., start when hands become contaminated after using the toilet, contact with a child’s excreta, coughing, sneezing, touching other people’s hands, and touching other contaminated surfaces. For example, a single gram of human feces can contain 10 million viruses and one million bacteria, and infant feces are particularly pathogenic [3,4]. Handwashing with soap works by removing bacteria and viruses before they can enter the body or spread to other people. Nevertheless, in many cases, handwashing is not possible. In such cases, the spread of infections can be prevented by the creation of antimicrobial (AM) and/or antiviral coatings, especially for high-traffic objects, such as switches in bathrooms, doorknobs, elevator buttons, sofas and chairs in public places such as cinemas, theaters, hotels, waiting rooms in hospitals, doctor’s offices, etc. From another point of view, environmental thinking obliges the use of bio-based and, if possible, bio-degradable materials that are non-toxic for humans via development of such types of products.

Essential oils (EOs) can be considered as key actives with AM properties, e.g., possessing antibacterial, antiviral, antiseptic, antioxidant, anti-parasitic, etc., activities, which have been reported in numerous publications [5,6,7]. In addition, Stefanakis et al. [8] emphasized that EOs can serve as a powerful means of reducing AMR. EOs (also called volatile oils) are renewable and can be obtained from different plant sources, such as leaves, buds, fruits and their shells, flowers, roots, seeds, etc. Unfortunately, EOs lose their activity over time due to their sensitivity to temperature, humidity, oxidation, UV-light, etc. Furthermore, their bioactivity may be affected by their natural volatility and low water solubility, resulting in a reduction in the overall efficiency of the antibacterial products containing free EOs [9].

To overcome these limitations, encapsulation into nanocarriers—such as polymeric sub-micro-/nanocapsules (NCs), liposomes, solid lipid nanoparticles, and mesoporous silica particles—has emerged as an effective strategy [10,11,12,13,14]. These systems offer controlled release, enhanced stability, improved bioavailability, and targeted delivery of EOs, allowing an enhanced AM efficacy at lower, safer doses [15]. Moreover, encapsulation can reduce adverse effects on mammalian cells and protect bioactive compounds from degradation or premature volatilization. Therefore, encapsulated EOs represent a promising class of natural, multifunctional AM agents suitable for applications in medicine, health care, cosmetics, food preservation, surface disinfection, etc.

Given the increasing demand for natural, biocompatible AM agents—especially in the context of antibiotic resistance—the development of EO-based nanomaterials offers a sustainable alternative to conventional antimicrobials. However, despite promising reports, comparative studies assessing the AM activity and cytotoxicity of different encapsulated EOs remain limited, particularly with respect to Gram-positive pathogens such as *Staphylococcus aureus*.

**Figure 1:**
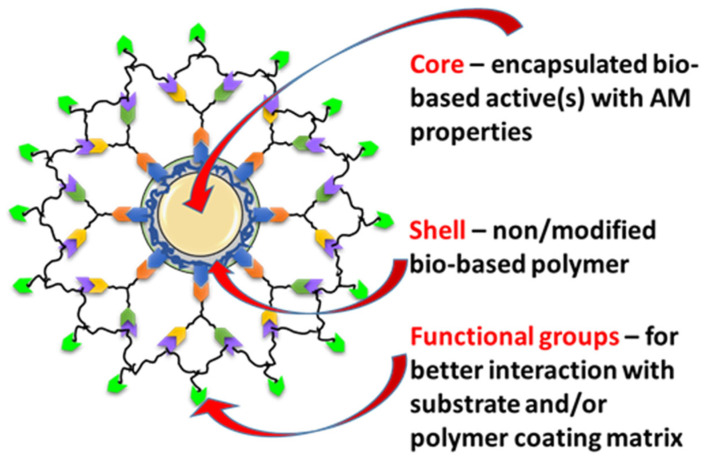
Structure of core–shell particles with low cytotoxicity and significant AM properties.

In this context, we developed various types of core-shell NCs with low cytotoxicity and significant antibacterial AM properties. The EOs selected for investigation, i.e., oregano (OO), eucalyptus (EuO), thyme (TO), and tea tree (TTO) oils, have been encapsulated as core materials within protecting bio-based and/or bio-compatible polymer shells (Figure 1).

Water insoluble protein zein and poly(methyl vinyl ether-co-maleic anhydride) (GZA) have been investigated as shell polymers, which have demonstrated good self-assembling properties via sedimentation in water. However, their direct use for essential oil encapsulation is often limited by insufficient colloidal stability, broad particle size distributions, and suboptimal interactions with hydrophobic EO components, which can negatively affect encapsulation efficiency and biological performance. To overcome these limitations, chemical modification of the polymer backbone represents an effective strategy to tailor physicochemical properties such as hydrophobicity, interfacial affinity, surface charge, and crosslinking ability. In this study, zein and GZA were chemically modified to enhance their compatibility with hydrophobic EOs, improve nanocapsule stability and size control, and ultimately reduce EO-associated cytotoxicity while preserving antibacterial efficacy.

Overall, the development of these NCs focused on the following three main aspects:

- The biogenic origin, biocompatibility, and/or biodegradability of the raw materials and synthesized products,
- high antibacterial activity combined with low cytotoxicity of NCs, and
- the simplicity and cost-effectiveness of their synthesis.

In addition, eco-design concepts are increasingly influencing research and development, especially as concerns about global warming and rising CO_2_ emissions drive the adoption of more environmentally conscious approaches [16]. Ensuring the long-term sustainability of advanced nanomaterials, such as nanocapsules (NCs), requires a comprehensive evaluation of their environmental impacts across the entire life cycle [17]. Life Cycle Assessment (LCA) provides a robust methodology for this purpose, examining all stages from raw material extraction and synthesis to use and final disposal.

Despite its strengths, applying LCA to nanomaterials remains challenging, primarily due to the limited availability of detailed Life Cycle Inventory (LCI) data for often poorly characterized materials. Within this framework, this study leverages LCA not only as a measurement tool but also as a strategy for guiding environmentally conscious NC synthesis.

Green synthesis approaches, as evaluated in this paper, employ natural substances such as plant extracts, essential oils, and biopolymers, reducing reliance on toxic chemicals and supporting renewable resources. Integrating life cycle thinking at early development stages allows for the identification of environmental hotspots, tracking of burdens throughout each synthesis step, and the generation of consistent, quantitative comparisons across multiple impact categories.

This rational design approach allows for systematic investigation of how polymer chemistry influences nanocarrier performance and supports the development of safer and more effective EO-based antimicrobial systems.

## 2. Materials and Methods

### 2.1. Chemicals and Reagents

AM actives, such as oregano (OO), eucalyptus (EuO), thyme (TO), and tea tree (TTO) essential oils, were purchased from V03 Trading GmbH (wesentlich.drogeriemarkt): (https://wesentlich.shop (accessed on 10 December 2025, Willich, Germany)). Shell-polymers: zein and GZA; solvents: 1,3-propanediol (PDO), ethanol, and acetone; surfactants: Tween 20, Tween 80, and oleic acid; modifiers for shell-polymers: vanillin (VA), dopamine hydrochloride, cinnamoyl chloride, dodecyl amine (DDA), 3-(glycidyloxypropyl)trimethoxysilane (EPTMS), as well as Oil Red O (ORO) were purchased from Aldrich (Darmstadt, Germany), while thiazolyl blue tetrazolium bromide (MTT), agar, and tryptic soy broth (TSB) were obtained from Sigma-Aldrich Ltd. (Poole, UK). RPMI 1640 medium with phenol red, penicillin/streptomycin, fetal bovine serum (FBS), phosphate-buffered saline (PBS), L-glutamine, and trypsin/ethylenediaminetetraacetic acid (EDTA) were obtained from Biowest (Nuaillé, France). 2-propanol (99.8%) and 1 M HCl solution were purchased from Merck KGaA (Calbiochem^®^, Darmstadt, Germany). These materials were used without additional purification. Water purified using an Omnia ultrapure water system from Stakpure GmbH was used as an anti-solvent.

### 2.2. Chemical Modification of Shell-Polymers and Synthesis of Core–Shell Particles

#### 2.2.1. Chemical Modification of Shell-Polymers

Chemical modification of shell-polymers was considered as a way to achieve better stability of NCs and introduce functional groups in particle shells. Chemical modification of protein zein was carried out using VA, dopamine hydrochloride, and cinnamoyl chloride. Modification was carried out directly in the 10 wt.% polymer solution in the PDO at 30 °C or 80 °C for 30 min. The following non-stoichiometric component ratios were used for modification: protein/modifier = 1:0.25, 1:0.5, 1:0.75 *w*/*w*.

**Figure 2:**
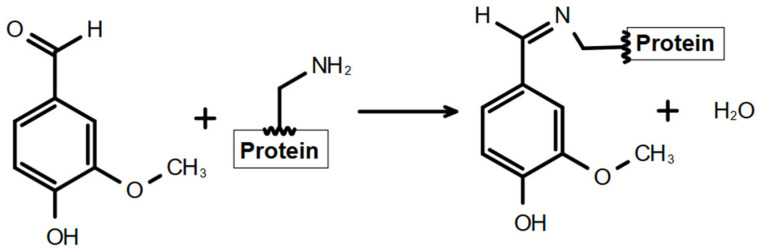
Scheme of the chemical modification of the protein macromolecule with VA.

Unfortunately, the modification products of zein with dopamine hydrochloride and cinnamoyl chloride were evaluated as unusable for encapsulation—the formed products had a very dark color and a pungent odor and, in addition, poor stability. Encapsulation experiments conducted with fresh-prepared modified polymer and after several days of preparation demonstrated a complete lack of reproducibility. Consequently, for all further experiments, zein modifications with VA (Figure 2) were exclusively used for NC synthesis and related investigations.

**Figure 3:**
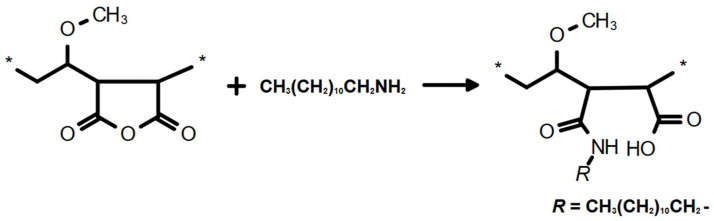
Scheme of the chemical modification of the GZA-copolymer with DDA (the monomer unit is marked between the symbols “*”).

**Figure 4:**
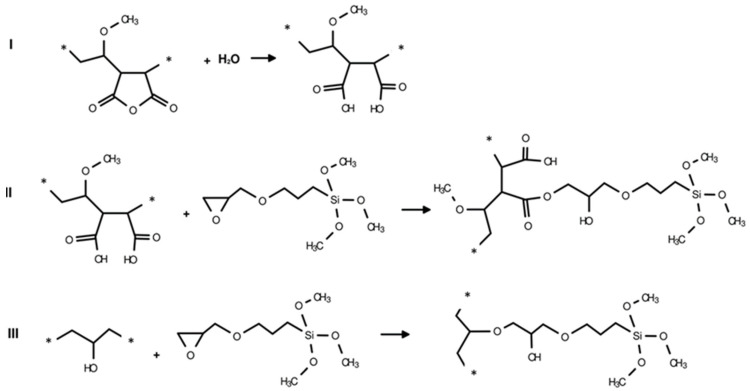
Scheme of the chemical modification of the GZA-copolymer with EPTMS, which takes place in steps I–III (the monomer unit is marked between the symbols “*”).

Modification of GZA-copolymer was carried out in 20 wt.% acetone solution with DDA or with EPTMS with a stoichiometric ratio of polymer/modifier = 1:0.25 at 120 °C for 2 h in a microwave reactor Monowave 400 (Anton Paar GmbH) (see Figure 3 and Figure 4, respectively).

The obtained modified polymers were used for synthesis and investigation of NCs.

#### 2.2.2. Core–Shell Particles Preparation—Design of Procedure and Preparation Method

Nanoprecipitation was selected as the base method for core–shell particles preparation due to its relative simplicity. The procedure of nanoencapsulation can be carried out in different ways, and factors such as the type and nature of the shell-building polymer and active to be encapsulated, metering and mixing speed, mixer type, temperature, nanoprecipitation via “solution-in-anti-solvent” or “anti-solvent-in-solution” route, etc., can influence the particle size distribution (PSD), polydispersity index (PDI) values, and stability of the resulting dispersions of NCs. To select the optimal nanoprecipitation conditions, preparations of so-called “empty” NCs, i.e., nanoparticles (NPs) without encapsulated EOs, were studied using different process conditions and routes for both non-modified polymers. The procedure providing the most promising Dynamic Light Scattering (DLS) results for both polymers (see Section 3.3) was selected as the most suitable one. It is noteworthy that the developed technology does not require any drying steps, providing a cost-efficient and easy-to-scale-up production process. Through this process, two different empty NP types (using either GZA or zein) were prepared and characterized. For this purpose, GZA and zein were solubilized in acetone and PDO and added at a constant flow rate using a precise peristaltic pump to the prepared-in-advance water solution of surfactants under agitation. After numerous trials, it was determined that for small dispersion batches (up to 500 g/batch), the Budde-Mix-mixer operated at 700–1200 rpm (revolutions per minute) at room temperature (RT) using the nanoprecipitation route “solution-in-anti-solvent” and a slow dropwise dosage of polymer solution (i.e., 0.5–1 mL/min), were the most suitable conditions for the formation of uniform NPs.

**Figure 5:**
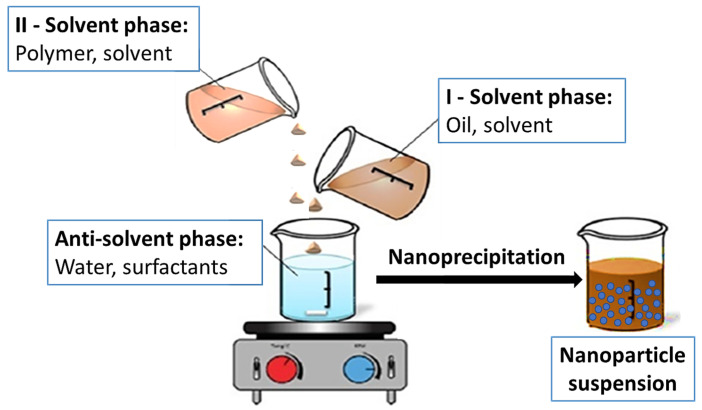
Scheme of the NC synthesis using the nanoprecipitation method “solution-in-anti-solvent”.

For the preparation of NCs containing EOs in the core, the following procedure was applied:

(1) A solution of an EO (I—solvent phase) was prepared by dissolving the oil in PDO+ ethanol or acetone using 10% of the amount of solvent(s) required for the synthesis (see Table 1);
(2) The anti-solvent phase was prepared by dissolving related surfactants in water at RT;
(3) A solution of either the modified or non-modified shell-building polymer (II—solvent phase) was prepared by dissolving the polymer in PDO+ Ethanol or acetone using the 90% of the amount of solvent(s) required for the synthesis (see Table 1);
(4) The solution of an EO (I—solvent phase) was added in (anti-solvent phase) with stirring at RT;
(5) Then, a solution of shell-building polymer (II—solvent phase) was added dropwise to the EO-in-water emulsion with continuous stirring at RT (Figure 5), resulting in the formation of a turbid dispersion;
(6) The obtained dispersion was then stabilized with continuous stirring for at least 20 min at RT.

In the case of NCs based on GZA-DDA modification as a shell-polymer, crosslinking of the obtained dispersions with EPTMS was additionally carried out under stirring at 70 °C for a during of 2 h to increase the stability of the shells. The EPTMS additional rate was maintained at 1 mL/min. The same crosslinking conditions were applied to NCs based on the GZA-EPTMS modification as the shell-polymer. The formulations used for the synthesis of GZA- and zein-based NCs are presented in Table 1.

All obtained dispersions were investigated for their stabilities (based on ζ-potential values) and PSDs using DLS measurements.

#### 2.2.3. Procedure for Determination of Encapsulation Efficiency of EOs

The effectiveness of EO encapsulation was determined as follows.

Fat-soluble azo dye Oil Red O (ORO) was used as the visualization dye. This dye is used primarily for staining neutral lipids and triglycerides in biological samples and is known for its ability to provide clear visual identification of lipids [18]. A solution of the dye in 2-propanol was prepared with a concentration of 2.0 × 10^−4^ mol/L. From this initial solution, new dye solutions with concentrations of 1.0 × 10^−4^ mol/L (solution 1, S1), 5.0 × 10^−5^ mol/L (S2), 2.5 × 10^−5^ mol/L (S3), 2.0 × 10^−5^ mol/L (S4), 1.0 × 10^−5^ mol/L (S5), 5.0 × 10^−6^ mol/L (S6), 2.5 × 10^−6^ mol/L (S7), 1.0 × 10^−6^ mol/L (S8), and 5.0 × 10^−7^ mol/L (S9) were prepared via dilution of appropriate aliquots with 2-propanol. 

**Figure 6:**
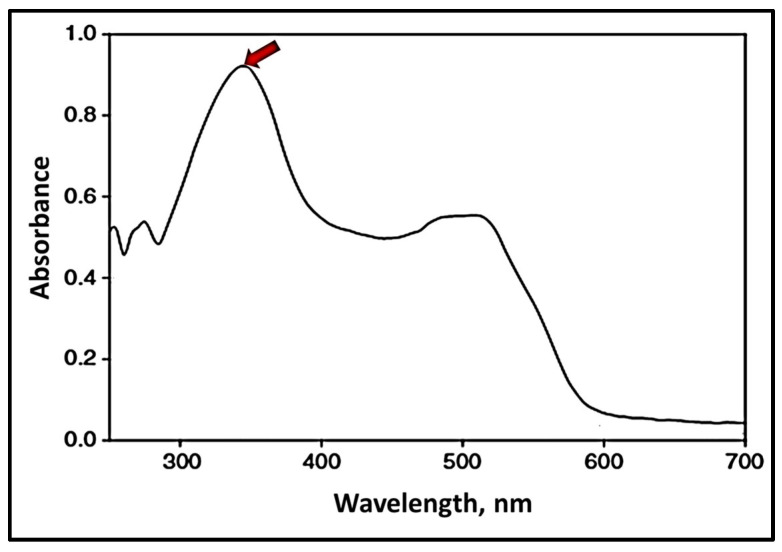
UV–vis spectrum of ORO in 2-propanol.

The obtained solutions of ORO were investigated using UV/VIS-spectroscopy (Specord 50, Analytik Jena GmbH+Co. KG, Jena, Germany) in the range of 250–700 nm. Quartz cuvettes with a path length of 1 cm were used for these investigations. The absorbance maximum in ORO spectra at 345 nm (Figure 6, red arrow) was selected as the base for quantitative determination of the dye.

For all investigated EOs, ORO was dissolved in oils at a concentration of 1 wt.%. Colored EOs were encapsulated according to the procedure described in Section 2.2.2. After preparation, the obtained dispersions were centrifuged for 20 min at 16,000 rpm using a Universal 320 centrifuge (Andreas Hettich GmbH & Co. KG, Tuttlingen, Germany). After that, the liquid phase of each system was transferred into a pre-weighed Petri dish and dried at RT to a constant mass. The dry residue was dissolved in 2-propanol. The concentration of the obtained solution (S10) was 1 wt.%.

For accurate quantification of the residual amount of non-encapsulated colored EOs, UV–vis spectra of these solutions (i.e., S10 for each system) were analyzed.

### 2.3. Equipment and Methods for Investigation of Polymers and Core–Shell Particles

#### 2.3.1. Investigation of Chemical Structures of Modified Shell Polymers Using FTIR

After chemical modification of shell-polymers, which was carried out in PDO or acetone solution, the resulting products were investigated using Fourier Transform Infrared (FTIR) spectroscopy (Nicolet iS10, Thermo Fisher Scientific Inc., Schwerte, Germany). The instrument was furnished with diamond Attenuated Total Reflection (ATR), and the spectra were acquired in the range of 600–4000 cm^–1^ using 32 scans per spectrum collected at a resolution of 0.4 cm^–1^ at RT.

After chemical modification, the modified polymer was isolated via sedimentation in water and filtration. After filtration, the filtered polymer was washed with ethanol and dryed at ambient conditions followed by post-drying in a vacuum oven at RT until a constant weight, then the modified polymer was re-dissolved. The cleaning procedure was repeated three times for better separation of the modified polymer from non-reacted fractions of low molecular chemicals and modifiers. After that, the dried polymer was investigated.

#### 2.3.2. Characterization of Dispersions of Core–Shell Particles Using DLS

The DLS measurements of the synthesized dispersions of the core–shell particles for the determination of the respective particle size, ζ-potential, and particle size distribution were carried out using the automated particle characterization system Zetasizer Nano ZS device (Malvern Panalytical GmbH, Kassel, Germany). Zetasizer Software Version 7.12 was used for analysis. Samples of the original dispersions of NCs were taken out and analyzed directly after synthesis and stabilization. Deionized water was used as the dispersion medium. The following settings were applied for the determination of particle size and PSD.

- Disposable plastic cuvettes (DTS 0012, polystyrene, minimum sample volume 1 mL).
- Measurement temperature, 25 °C; equilibration time, 120 s; viscosity, 0.8872 cP; refraction index (RI), 1.330.
- Mark–Houwink parameters: a-parameter, 0.428; K-parameter, 7.67 × 10^–5^ cm^2^/s.
- Measurement angle, 173°; default non-invasive backscatter (NIBS).
- Number of runs per measurement, 11; run duration, 10 s; number of measurements, 3; delay between measurements, 0 s.
- Analysis model: multiple narrow modes (high resolution).

The following parameters were used for measurement of the ζ-potential.

- Disposable folded capillary cell (DTS 1070, polystyrene, minimum sample volume 0.75 mL).
- Measurement temperature, 25 °C; equilibration time, 120 s; viscosity, 0.8872 cP; RI, 1.330; dielectric constant, 78.5.
- Smoluchowski model: F (Ka) value, 1.50.
- Automated selection of attenuation and voltage (based on the measured conductivity of the samples; default is enabled).
- Number of runs per measurement, 10; number of measurements, 3; delay between measurements, 0 s.
- Analysis model: auto mode (default setting); in this mode, the software determines the most suitable type of measurement to perform after measuring the sample conductivity.

### 2.4. Evaluation of Antibacterial Activity

The antibacterial activities of the raw EOs as well as the as-prepared core–shell particles loaded with EOs were assessed by determining their minimum inhibitory concentration (MIC) and minimum bactericidal concentration (MBC) values. MIC values were obtained using the macro-dilution method, while MBC values were measured through the colony-counting method, in accordance with the CLSI M07-A9 and M26-A protocols, respectively [19,20].

*Bacteria Culture*: *Staphylococcus aureus* (*S. aureus*) bacteria strain ATCC 25923 was used as the model Gram (+) bacterial strain. *S. aureus* bacteria were grown in tryptic soy broth (TSB) under aerobic conditions with continuous shaking at approximately 200 rpm using a Stuart SI500 orbital shaker (Bibby Scientific Ltd., Staffordshire, UK) at 37 °C for 16 h. After incubation, the bacterial suspension was diluted with TSB medium to achieve a concentration equal to 0.5 McFarland Standard, corresponding to approximately 10^8^ colony-forming units per milliliter (CFU/mL).

*Determination of MIC and MBC values*: *S. aureus* (at a final concentration of 10^5^ CFU/mL) were inoculated into 10 mL tubes, followed by the addition of 2 mL aqueous nanoparticles dispersions or aqueous solutions of EOs of various concentrations (1–500 μg/mL). Untreated bacterial cultures served as controls. The MIC was defined as the lowest concentration of the encapsulated or not EO that inhibited visible bacterial growth after a 24 h incubation time at 37 °C. To determine the MBC, 50 μL aliquots of the treated bacterial suspensions from the MIC assay were collected—specifically from the MIC concentration tube and from three higher concentrations—and plated onto LB agar plates. The plates were incubated for 18 h at 37 °C, then the number of colonies was counted. The MBC was identified in terms of the lowest concentration at which 99.9% of the initial bacterial population was eradicated. All tests were performed in triplicate.

### 2.5. In Vitro Assessment of Cytotoxicity

The cytotoxic effects of the raw EOs as well as the as-prepared core–shell nanoparticles containing them were assessed using human embryonic kidney (HEK293) cells, as well as human prostate cancer cell lines PC3 and DU145, using the standard MTT assay.

*Cell Culture Conditions:* Human embryonic kidney (HEK293) cells and prostate cancer cell lines (PC3 and DU145) were cultured in RPMI 1640 medium supplemented with 10% fetal bovine serum (FBS), 2 mM L-glutamine, and antibiotics (penicillin at 100 U/mL and streptomycin at 100 μg/mL). Cultures were maintained at 37 °C in a humidified atmosphere containing 5% CO_2_.

*Cell Seeding and Treatment*: Cells were detached using a trypsin–EDTA solution (0.05% *w*/*v* trypsin and 0.02% *w*/*v* EDTA) and sub-cultured biweekly. For the assay, 10^4^ cells per well were seeded into 96-well plates and allowed to adhere and grow for 24 h at 37 °C in a humidified atmosphere containing 5% CO_2_. Subsequently, cells were exposed to varying concentrations of the encapsulated or unencapsulated EOs for 3 h. Following treatment, the supernatants were discarded, replaced with fresh, complete medium, and incubated for 24 h.

*MTT Assay Procedure:* Cell viability was assessed using the MTT assay, which measures mitochondrial metabolic activity. Thus, after the 24 h incubation period, the medium was replaced with 100 μL of MTT solution (10 μg/mL in complete medium), and cells were incubated for 3 h at 37 °C in a 5% CO_2_ atmosphere. Following incubation, the MTT solution was carefully removed, and the resulting formazan crystals were dissolved in 100 μL of 2-propanol per well. Absorbance was measured at 540 nm using an Infinite M200 microplate reader (Tecan Group Ltd., Männedorf, Switzerland). Blank values from wells containing only 2-propanol (without cells) were subtracted from all readings.

*Statistical Analysis*: Cell viability was expressed as a percentage relative to untreated control cells. All respective data are represented as the mean ± standard deviation (SD) from at least three independent experiments, each performed in eight replicates per concentration. Statistical analysis was performed using Student’s paired two-tailed *t*-test to evaluate differences in MTT cytotoxicity between each EO and its encapsulated counterpart. Additionally, independent *t*-tests were used for comparisons between unencapsulated and encapsulated forms of each EO. The statistical significance follows the assignment: * *p* < 0.05, ** *p* < 0.01, *** *p* < 0.001, **** *p* < 0.0001, and ns (not significant: *p* > 0.05).

### 2.6. LCA Methodology Applied to Developed Bio-Based NCs

LCA application is grounded in internationally recognized standards, according to ISO 14044:2006 [21], which provides structured procedures to assess and compare environmental impacts throughout the product’s life span. Most existing LCA studies on nanomaterials adopt a cradle-to-gate approach, excluding the end-of-life stage due to limited data and methodological challenges [17]. This study follows the same boundary, focusing on production-related impacts, as use phase and end-of-life stages for nanomaterials remain highly uncertain and difficult to model accurately. This study aims to address this shortcoming by providing laboratory-scale LCI data along with key findings from the LCA. The functional unit and the systems introduced in SimaPro 10.2.0.1 software are described in Table 2.

## 3. Results and Discussion

### 3.1. FTIR Results for Shell-Building Polymers

To confirm that chemical modification of shell-polymers took place, FTIR-spectra of the original and modified polymers were compared. The appearance of new absorption bands confirming the formation of new functional groups due to chemical modification and respective reduction/disappearance of bands characteristic for reactive functional groups of unmodified polymers in FTIR-spectra of modified polymers were analyzed for this purpose.

**Figure 7:**
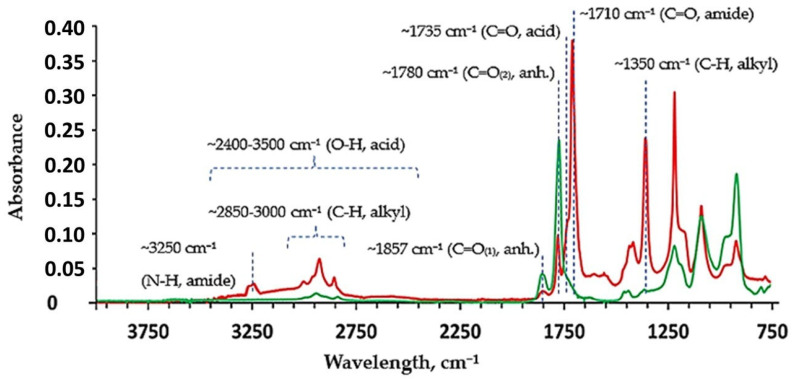
FTIR spectra of DDA-modified GZA copolymer (red) and unmodified GZA copolymer (green), showing spectral changes associated with the ring-opening modification of anhydride groups by DDA.

The spectrum of the DDA-modified GZA-copolymer (Figure 7, red) compared to unmodified GZA-copolymer (Figure 7, green) showed the appearance of new bands at ~1710 cm^−1^ and ~3250 cm^−1^ corresponding to the C=O and N-H stretching vibrations of a carboxylic acid amide, respectively.

This was accompanied by the occurrence of a broad band in the range of ~2400–3500 cm^−1^ characteristic of O-H stretch, which can be allocated to the formation of carboxylic groups. Furthermore, an increase of the band intensities at ~2850–3000 cm^−1^ and ~1350 cm^–1^, typical for C-H stretching and bending vibrations, respectively, was observed due to the incorporation of alkyl chains of DDA in the structure of the polymer. At the same time, the bands associated with the C=O stretching vibrations of carboxylic acid anhydride (i.e., (C=O)-O-(C=O) groups) at ~1857 cm^−1^ and 1780 cm^−1^ were also affected in comparison with the unmodified sample. In addition to the reduction in the intensities of these bands, the occurrence of a C=O stretching signal in form of a shoulder at ~1735 cm^−1^, characteristic of carboxylic groups, was observed. These changes confirm the nucleophilic ring-opening reaction of anhydride moieties with DDA resulting in the formation of carboxylic and amide groups [22].

**Figure 8:**
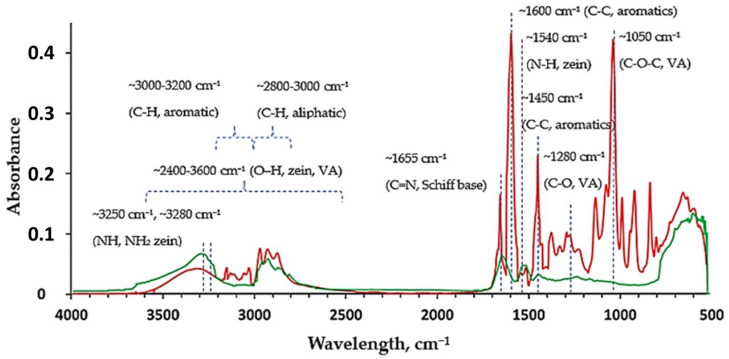
FTIR spectra of VA-modified (red) and unmodified zein (green).

The FTIR spectrum of the VA-modified zein shows distinct features that confirm successful chemical modification (Figure 8, red). A clear absorption band appears at 1655 cm^−1^, corresponding to the C=N stretching vibration of an imine group. However, this band overlaps with the region characteristic for C=O-stretching in zein (band at ~1650 cm^−1^). Additionally, the intensities of overlapping signals at ~3250 cm^−1^ and ~3280 cm^−1^, associated with the stretching of NH and NH_2_ groups of zein, respectively, as well as of the band at ~1540 cm^−1^, characteristic of N-H bending, decreased in comparison with the unmodified sample (Figure 8, green) [23]. These findings indicate the formation of Schiff base structures through the reaction between the aldehyde group of VA and amine groups of zein. The bands corresponding to aromatic moieties of VA in modified zein appearing at ~1600 cm^−1^ and ~1450 cm^−1^ (C-C stretches in ring) and ~3000–3200 cm^−1^ (C-H stretch) possess obviously higher intensities compared to the respective signals of unmodified zein and act as additional confirmation of chemical modification. The low intensity of aromatic bands of unmodified zein is related to the relatively low content of aromatic amino acids in its structure. The bands at ~1280 cm^−1^ and ~1050 cm^−1^ are attributed to C–O and C–O–C stretching vibrations, respectively, which reflect contributions from the ether groups introduced by VA. These spectral changes confirm the successful conjugation of VA to zein via imine bond formation [24].

Since the EPTMS-modified GZA copolymer is very sensitive to moisture, precipitation in water caused it to crosslink. This is why FTIR studies were not performed for this polymer modification. Confirmation that chemical modification has occurred is the color change of the reactants (the solution of GZA copolymer and EPTMS) after mixing. Initially, the reaction mixture turned from colorless to pale pink, and after 2 h in a microwave reactor at 120 °C, it acquired an intense pink color.

### 3.2. Determination of Encapsulation Efficiency of Essential Oils

Since ORO is insoluble in water at room temperature but has very good solubility in oils and intense absorption in the UV–vis range, it is a very good candidate for determining the encapsulation efficiency of EOs when this dye is dissolved in them.

According to the Beer–Lambert Law, the absorbance is directly proportional to the concentration of the substance in the solution. Therefore, a sample’s concentration can also be determined using UV–visible spectroscopy.

The Beer–Lambert Law can be expressed in the form of Equation (1):

(1) A=ε·c·l,

where A = absorbance,

l = optical path length of the cell or cuvette or sample holder (cm),
c = concentration of the solution (mol/L),
ε = molar absorptivity of the compound or molecule in solution, which is constant for a particular substance at a particular wavelength (L/(mol·cm)).

Following the Beer–Lambert Law, the plot of absorbance versus concentration should be linear if the absorbance of a series of sample solutions with known concentrations is measured and plotted against equivalent concentrations. This graph is a calibration graph, which can be used for the determination of compound concentration in a solution with known absorbance.

**Figure 9:**
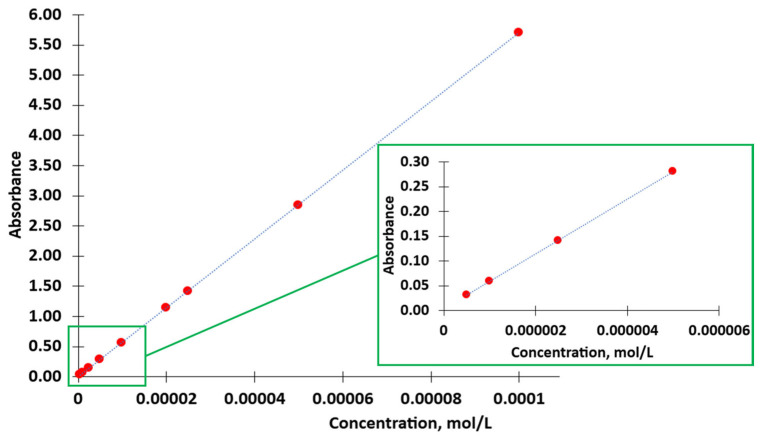
Dependence of absorbance from concentration of ORO in 2-propanol.

Since in UV research, in addition to the substance to be investigated and the thickness of the absorbing layer, the solvent also remains unchanged, possible influences of the solvent on the intensity and shape of the signal can be neglected. Therefore, we accept without a doubt that there is a linear relationship between absorbance and concentration. As mentioned in Section 2.2.3., the absorption band at 345 nm was used as a base for the determination of dye concentration. Figure 9 presents a calibration graph of Absorbance/Concentration for solutions of ORO in 2-propanol.

As can be seen in the calibration graph, the deviation of the experimental points from the linear dependence is very insignificant, which allows us to determine the concentration of the dye in the S10 solution quite accurately.

The following amounts of the EOs and respective amounts of the solved dye (i.e., 1 wt.% in an EO) were used for encapsulation in different shell polymers to determine the encapsulation efficiency:

- zein-based NCs: 20 g of oil (0.2 g or 4.896 × 10^−4^ mol ORO) per 1000 mL of dispersion,
- GZA-based NCs: 50 g of oil (0.5 g or 1.22 × 10^−3^ mol ORO) per 1000 mL of dispersion.

**Figure 10:**
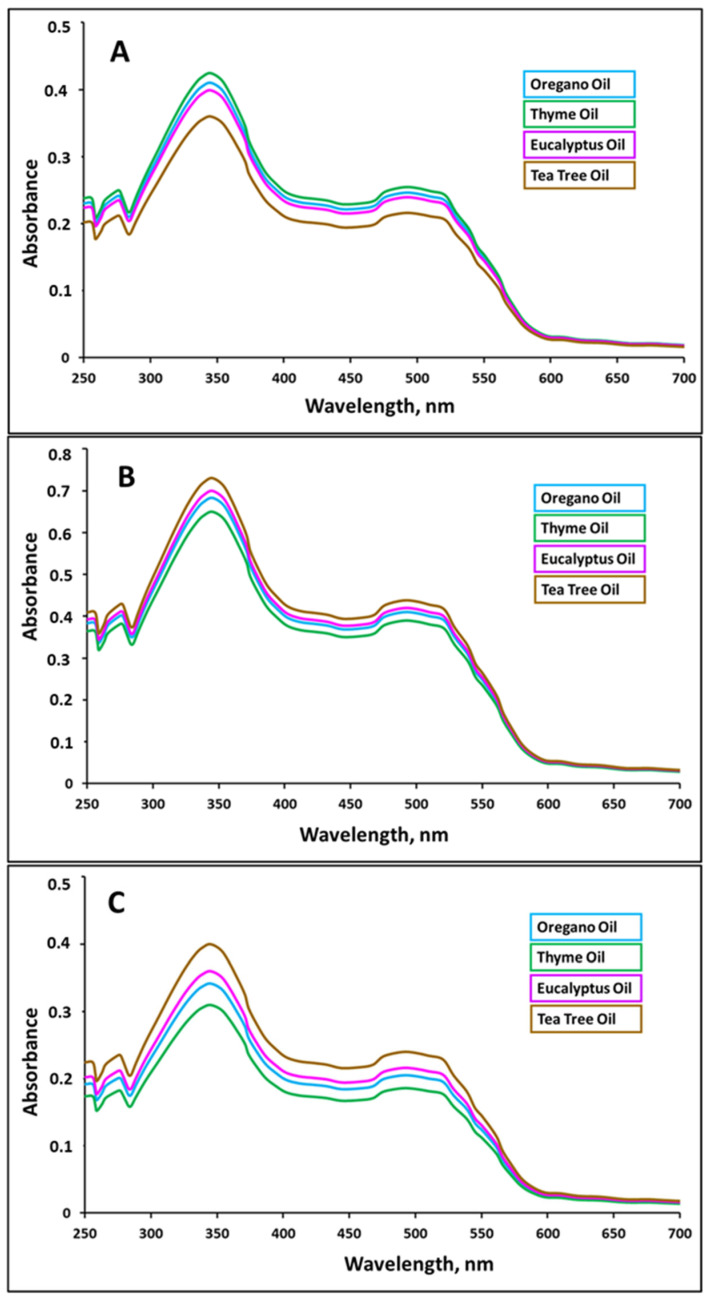
Absorbance for S10 solutions obtained after encapsulation of different EOs in VA-modified zein (**A**), EPTMS-modified GZA (**B**), and DDA-modified GZA (**C**).

The encapsulation efficiency was determined as follows. First, the absorbance of the ORO in the obtained S10 solution was determined. Figure 10 presents the absorbance for S10 solutions obtained after encapsulation of different EOs in VA-modified zein (Figure 10A), EPTMS-modified GZA (Figure 10B), and DDA-modified GZA (Figure 10C).

Then, the concentration of the dye in the S10 solution was calculated according to the Beer–Lambert equation using the obtained absorbance value at 345 nm and the calibration graph. This value is proportional to the amount of oil that was not encapsulated. Finally, the encapsulation efficacy was calculated using Equation (2):

(2) $$E_{ef}=\left(1-\frac{\chi_{f}}{\chi_{i}}\right)\times 100\%$$

where $\chi_{f}$ is the concentration of ORO in related S10 solution and $\chi_{i}$ is the initial concentration of ORO per 1000 mL dispersion (i.e., 4.896 × 10^−4^ mol/L for zein-based NCs and 1.22 × 10^−3^ mol/L for GZA-based NCs).

The determined concentration and encapsulation efficiency values for different NC types are presented in Table 3.

The obtained results demonstrate that the procedure developed for synthesis of NCs provides a high encapsulation efficiency. The highest efficiency values were observed for the DDA-modified GZA shell-polymer. This can be attributed to the better affinity of this polymer to EOs due to hydrophobic dodecyl moieties of DDA.

### 3.3. Influence of Chemical Modification of Shell-Polymer on Size and Stability of EO-Filled NCs

**Figure 11:**
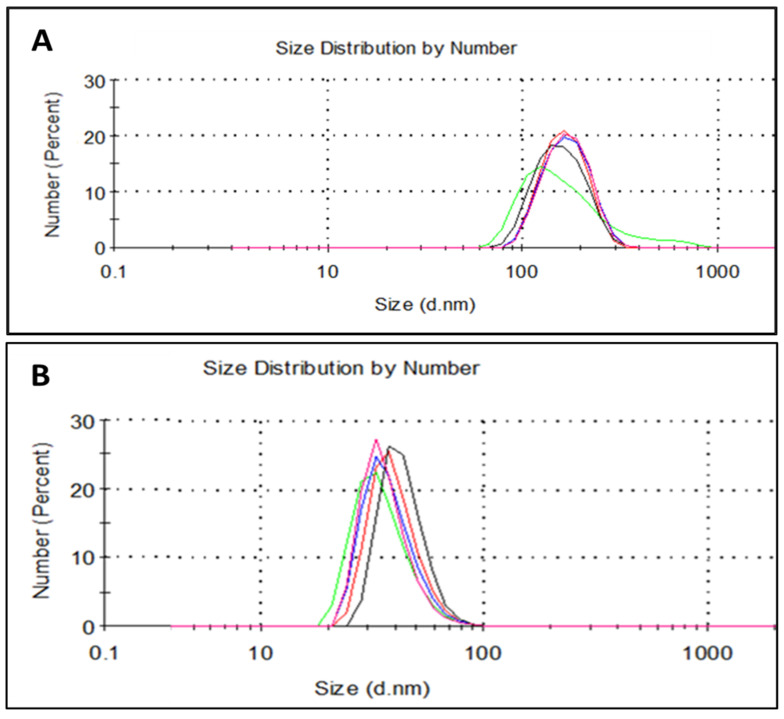
Particles size distribution by number for “empty” NCs based on zein (**A**) and GZA (**B**). Each figure represents the PSD of the same sample measured 5 times at 5 min intervals in the following sequence: green, red, blue, pink, black.

Considering that the nanoencapsulation procedure was performed based on a nanoprecipitation route developed and optimized for the preparation of the “empty” NCs, both “empty” NCs and those containing different EOs—based on both non-modified and chemically modified shell-building polymers—were characterized and compared. As shown in Figure 11, “empty” NCs prepared from non-modified zein and GZA polymers according to the developed procedure (see Section 2.2.2) demonstrated quite uniform and narrow monomodal particles size distributions, especially in the case of GZA.

It is worthy to note that “empty” GZA-NCs (Figure 11B) of obviously smaller average size and higher dispersion stability (according to ζ-potential values) were obtained compared to those based on zein (Figure 11A), whereas similar polydispersity was achieved for both types of “empty” NCs. Zein-NPs resulted in an average particle size of ~170 nm, polydispersity index (PDI) of 0.128, and ζ-potential of −6.9 mV, whereas GZA-polymer-formed NPs had a particle size of ~40 nm, PDI of 0.120, and ζ-potential of −32.5 mV.

**Figure 12:**
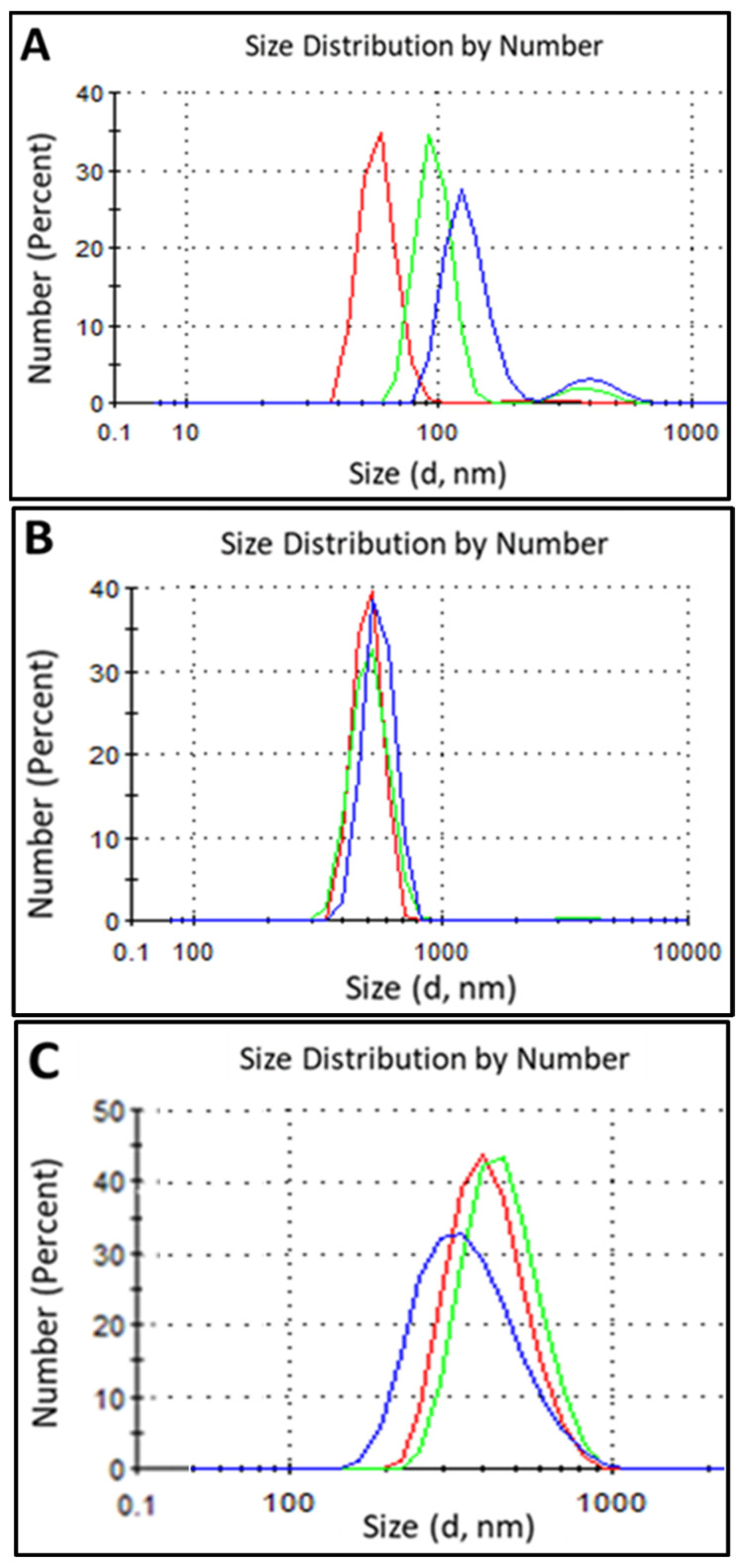
PSD by number for NCs filled with oregano oil based on non-modified zein (**A**), VA-modified zein (**B**), and DDA-modified GZA polymer crosslinked with EPTMS (**C**). Each figure represents the PSD of the same sample measured 3 times at 5 min intervals in the following sequence: green, red, blue.

Despite application of the most suitable nanoprecipitation route, increases in particles size and PDI-values and decreases in the dispersions’ stability were observed based on the synthesis of EO-filled NCs. As can be seen in the example of NCs based on nonmodified zein containing oregano oil (Figure 12A), bimodal PSD with average particle sizes of ~50 nm and ~200 nm with PDI of 0.514 and ζ-potential of 0.3 mV were obtained. Unfortunately, swelling and partial dissolving of shell-polymers in encapsulated actives was observed for EO-filled NCs based on nonmodified GZA as shell-building polymer. Therefore, no DLS-results for this NC-type are presented here.

For improvement of PSD, PDI, and dispersion stability, modified shell-building polymers were used for NC synthesis. When vanillin-modified zein (zein-VA) was used as a shell polymer, formation of larger but more uniform NCs was observed compared to those based on unmodified polymer. This can be explained by the ability of small aromatic moieties of VA to self-assemble via π–π-stacking interactions [25]. As shown in Figure 12 (compare A and B), double-peak PSD for NCs loaded with oregano oil based on non-modified zein changed to a single-peak PSD when using the VA-modified protein as the shell polymer. At the same time, improvement in the PDI and ζ-potential values for dispersions of NCs based on zein-VA was observed: PDI changed from 0.519 to 0.288 and ζ-potential from 0.2 mV to −6.25 mV for filled NCs with non-modified and VA-modified zein, respectively. Modification of GZA-polymer with DDA or EPTMS resulted not only in improved PSD and PDI values for synthesized NCs but also in higher stability of the shell, i.e., any swelling or dissolution of the shell was observed. Oregano oil-loaded NCs based on DDA-modified GZA resulted in monomodal PSD with average particle size of ~129 nm, narrow PDI of 0.107, and characteristic for a stable dispersion value of ζ-potential of −29.5 mV. Further improvement in DLS results was observed when these NCs were crosslinked with EPTMS (Figure 12C), i.e., uniform PSD with an average particle size of ~193 nm, PDI of 0.107, and ζ-potential of −30.1 mV. It can be noted that an increase in average particle size was expected because of the incorporation of the crosslinking agent within the structure of NC-shells.

**Figure 13:**
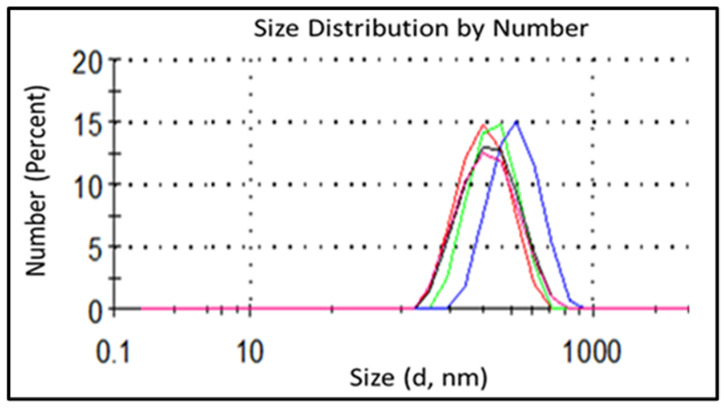
PSD by number for crosslinked NCs based on EPTMS-modified GZA and oregano oil measured 5 times at 5 min intervals in the following sequence: green, red, blue, pink, black.

The use of EPTMS-modified GZA as the shell polymer resulted in a notable effect on the size of the loaded NCs. In the case of this polymer modification, a crosslinking moiety is incorporated into the structure of the polymer chain prior to nanoprecipitation and formation of NCs. Immediately after the formation and stabilization procedures, i.e., before crosslinking, the NCs exhibited the following characteristics: average particle size ~382 nm, PDI 0.223, and ζ-potential −32.8 mV. After the crosslinking step at 70 °C for 2 h, the NCs were uniform, with a larger average particle size of ~472 nm, a smaller PDI of 0.201, and a similar ζ-potential of −33.1 mV (Figure 13).

As shown in Figure 12C and Figure 13, chemical modification of the same shell polymer (i.e., GZA) with different modifiers, namely DDA and EPTMS, caused a significant difference in the average sizes of the formed NCs. This could be explained as follows: DDA-modification of GZA forms macromolecules with markedly higher affinity to essential oils (i.e., in terms of polarity) due to the presence of long hydrophobic moieties, leading to the formation of small and uniform NCs. In contrast, EPTMS-modification reduces the affinity of the macromolecules to the oils compared with the DDA-modified polymer, resulting in the formation of larger NCs with higher PDI-value. Following the crosslinking step, the size of NCs increases in both cases due to the formation of a secondary shell, but the PDI- and ζ-potential-values remain largely unaffected.

For NCs encapsulating eucalyptus (EuO), thyme (TO), and tea tree oil (TTO) using VA-modified zein, DDA- and EPTMS-modified GZA, the same encapsulation procedure demonstrated similar trends to those observed for oregano oil, used here as a representative EO. The maximal deviation in particle size, PDI-, and ζ-potential-values was 9.26%, which can be attributed to differences in the nature of each EO.

In general, it can be concluded that the developed encapsulation procedure is well suited for synthesizing uniform and stable NCs loaded with EOs. Chemical modification and crosslinking of shell polymer positively influence NC stability, PSD and PDI-values, as well as dispersion stability. It should be also noted that DDA- and EPTMS-modified GZA formed more stable dispersions with smaller particles in comparison with VA-modified zein, and this trend was consistent for all NCs containing EuO, TO, and TTO.

**Figure 14:**
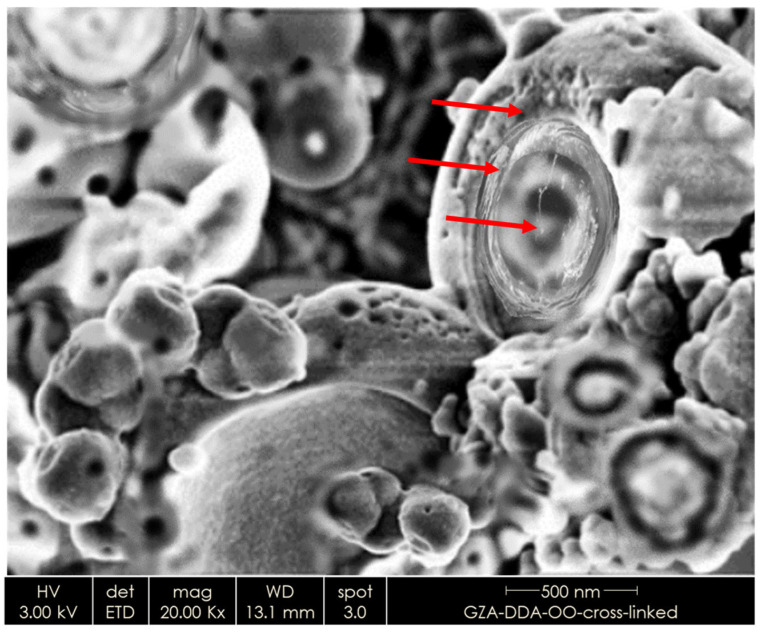
Representative SEM image (×20,000) of bio-based NCs based on DDA-modified GZA filled with OO and cross-linked with EPTMS, dried from water dispersions on dense paper towel.

The formation of core-shell structure can be confirmed by SEM analysis. Figure 14 presents a SEM image of bio-based NCs based on DDA-modified GZA filled with OO and cross-linked with EPTMS. The formation of core and two shells—modified GZA and polysilicate (by crosslinking with silane) can be observed (as indicated by the red arrows from bottom to top).

### 3.4. Cytotoxicity of Developed Bio-Based Core–Shell NCs

The cytotoxic effects of the raw EOs, i.e., OO, TO, EuO, and TTO, and their corresponding encapsulated form into NCs based on VA-modified zein (zein-VA), NCs based on EPTMS-modified GZA-polymer (GZA-EPTMS), or NCs based on DDA-modified GZA-polymer (GZA-DDA), were assessed on HEK293, DU145, and PC3 cells using MTT assays after 24 h of exposure.

**Figure 15:**
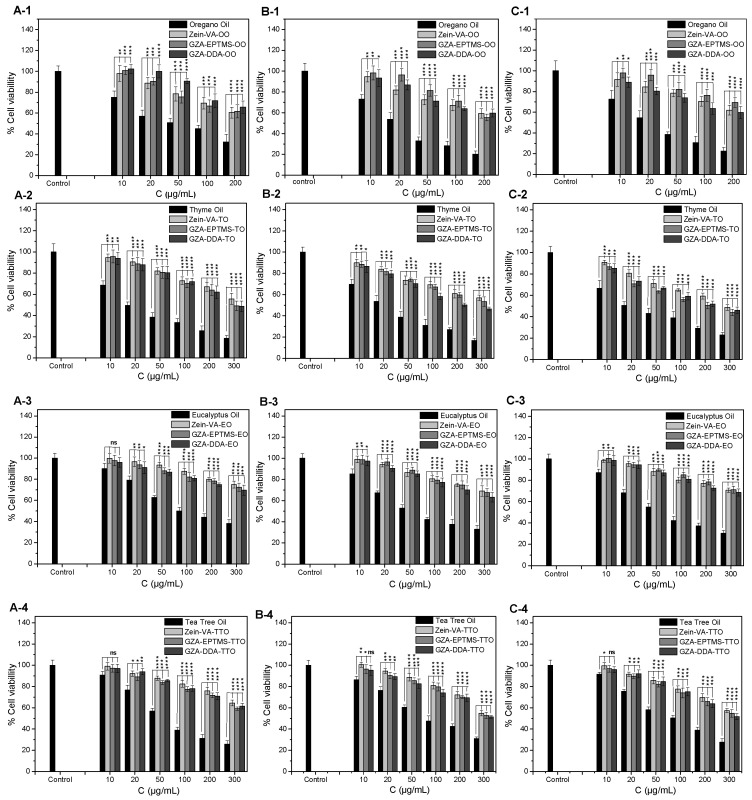
Comparative cytotoxicity of raw EOs, i.e., (**1**) OO, (**2**) TO, (**3**) EuO, and (**4**) TTO, and their corresponding encapsulated form into NCs based on Zein-VA, GZA-EPTMS, or GZA-DDA on HEK293 (**A**), DU145 (**B**), and PC3 (**C**) cells after 24 h of incubation, as assessed using MTT assays. Data are presented as mean ± standard deviation (SD) from eight replicates, obtained from at least three independent experiments. Statistical significance was determined using Student’s paired two-tailed *t*-test comparing each EO with its corresponding encapsulated counterpart. Significance levels are indicated as follows: * *p* < 0.05, ** *p* < 0.01, *** *p* < 0.001, **** *p* < 0.0001, and ns, not significant (*p* > 0.5).

As shown in Figure 15, raw EOs induced a significant reduction in cell viability in a dose-dependent manner. OO and TO exhibited the highest cytotoxicity (EC_50_ ≈ 20 μg/mL), against all tested cell lines, compared to EuO and TTO (EC_50_ ≈ 50 μg/mL). These findings are consistent with previous studies showing that free EOs, particularly those rich in thymol and carvacrol (as in OO and TO), possess strong cytotoxic activity due to their membrane-disruptive effects and induction of oxidative stress [26,27,28,29,30,31,32].

On the other hand, upon encapsulation, a statistically significant reduction in cytotoxicity was observed in all cases (* *p* < 0.05 to **** *p* < 0.0001), indicating that encapsulation enhances their biocompatibility probably by modulating the release of active components. Specifically, encapsulated OO or TO were found to be sub-toxic (survival ~60%) at the higher tested concentrations (200–300 μg/mL), revealing that both oils retained moderate activity against cell lines, suggesting partial preservation of their bioactive properties.

On the contrary, encapsulated EuO and TTO displayed minimal cytotoxicity (survival ~80% at the concentration of 300 μg/mL). This behavior could be attributed to differences in capsule permeability, oil–polymer interaction, or release kinetics. Similar observations were reported in the literature in which encapsulating EOs such as cardamon, babchi, OO, and TO in polymeric nanocarriers reduced their toxicity toward mammalian cells while retaining AM activity [28,29,30,31,32]. Therefore, nanoencapsulation effectively modulates the cytotoxic profile of EOs, enhancing their safety profile.

### 3.5. Antibacterial Activity of Bio-Based NCs Encapsulating EOs

The antibacterial efficacy of the NCs encapsulating EOs was assessed through the determination of their MIC and MBC values against Gram (+) *S. aureus* bacteria using the broth macro-dilution and colony-counting methods, respectively, following the CLSI M07-A9 and M26-A protocols, respectively. For comparison, raw EOs were also studied under the same conditions. As shown in Table 4, among the raw EOs tested, TO and OO demonstrated the highest antibacterial activity. Both exhibited remarkably low MIC values of 2 µg/mL, indicating their strong inhibitory effects. Additionally, TO achieved a bactericidal effect at the same concentration (MBC = 2 µg/mL), whereas OO required a slightly higher concentration (MBC = 5 µg/mL), confirming its potent but slightly less effective bactericidal action. TTO showed moderate antibacterial performance, with MIC and MBC values of 5 µg/mL and 50 µg/mL, respectively. In contrast, EuO displayed the weakest activity among the raw EOs, with both MIC and MBC values at 50 µg/mL, suggesting limited effectiveness against *S. aureus* in its unencapsulated form. These findings are consistent with existing literature, which reports that OO and TO possess strong antibacterial activity against *S. aureus*, mainly attributed to their high concentrations of phenolic compounds such as carvacrol and thymol. These compounds are known to disrupt bacterial cell membranes, increase permeability, and interfere with essential intracellular processes, ultimately leading to cell death [33,34,35]. TTO has been widely documented to exhibit broad-spectrum AM properties, including activity against methicillin-resistant *S. aureus* (MRSA) strains [36,37], while EuO exhibits moderate activity and is often used as a supportive AM agent, particularly due to its anti-inflammatory and mucolytic properties in respiratory applications [38].

On the other hand, the NC formulations, designed to enhance stability and reduce cytotoxicity of EOs, showed varied performance depending on the type of carrier matrix and surface modification used. For OO, NCs based on GZA-EPTMS exhibited relatively good antibacterial activity, with MIC and MBC values of 20 µg/mL and 50 µg/mL, respectively, being still significantly less effective than the raw oil but maintaining some potency. Similarly, GZA-DDA NCs containing OO demonstrated a MIC and MBC value of 100 µg/mL, indicating reduced efficacy. Zein-VA NCs with OO were less effective, with a high MBC value of 500 µg/mL, despite a modest MIC value of 20 µg/mL. In the case of TO, EPTMS- and DDA-modified GZA-based NCs showed MIC values of 20 µg/mL and MBC values of 200 µg/mL. However, zein-VA NCs were significantly less effective, requiring 500 µg/mL to achieve both MIC and MBC. For EuO, only GZA-EPTMS NCs showed measurable antibacterial activity (MIC = 30 µg/mL, MBC = 200 µg/mL), while both zein-VA and GZA-DDA NCs had MIC and MBC values above 500 µg/mL, suggesting negligible effectiveness. A similar pattern was observed with NCs containing TTO: only the GZA- EPTMS formulation showed moderate antibacterial activity (MIC = 30 µg/mL, MBC = 200 µg/mL), while the others were ineffective.

Overall, while raw EOs, particularly TO and OO, exhibited strong antibacterial properties, nanoencapsulation generally led to a reduction in AM activity. Similar observations have been reported in the literature; although the encapsulation of EOs into nanocarriers typically enhances their stability, bioavailability, and shelf life, encapsulated EOs often demonstrate lower AM efficacy than their free counterparts. This reduction is commonly attributed to the delayed or incomplete release of active components, particularly in systems incorporating tight polymeric matrices [39] or rigid inorganic carriers such as mesoporous silica [40]. For instance, thymol encapsulated in gelatin-based NPs showed reduced activity against *S. aureus* due to limited diffusion from the matrix and strong interactions with the polymeric carrier [41].

Among the various encapsulated formulations evaluated, nanocarriers based on EPTMS-modified GZA-polymer consistently outperformed other types, suggesting that polymer structure and surface modification are critical not only for reducing the toxicity of EOs (as previously noted) but also for preserving their antibacterial efficacy. These findings underscore the importance of thoughtful nanocarrier design, where a careful balance must be achieved between controlled release, minimized toxicity, and efficient delivery of bioactive EO components to target pathogens. Nevertheless, further studies are required to more clearly elucidate the underlying antibacterial mechanisms, as well as to systematically assess the influence of EO release kinetics and nanocapsule size on antibacterial efficacy and safety. 

### 3.6. LCA of Developed Bio-Based NCs

#### 3.6.1. Goal and Scope Definition

The goal of this study is to compare two NC formulations across four scenarios, each involving different EOs and the presence or absence of crosslinking agent to assess their environmental performance using LCA, with a focus on how the oil type and crosslinking process may affect its carbon footprint. A sensitivity analysis was included, selecting energy consumption as a key parameter given the notable differences between the GZA- and zein-based NC formulations. This analysis also highlights the role of crosslinking versus non-crosslinking processes, which influence environmental impact.

**Figure 16:**
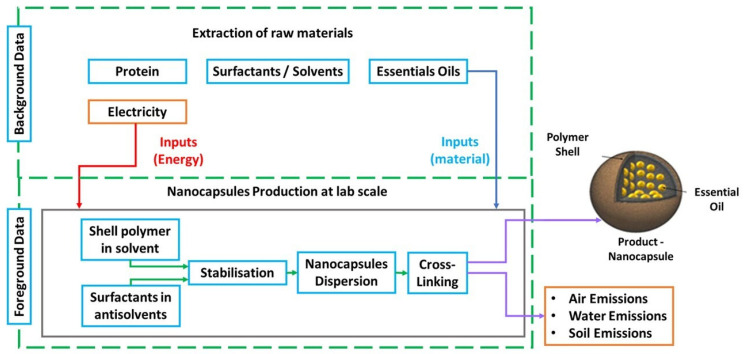
System boundaries of NC-production at laboratory scale.

The system boundaries defined as cradle-to-gate for the assessment are shown in Figure 16. The functional unit refers to the production of 100 mL of NC formulation in Europe (without considering the antibacterial activity); the total amount belongs to the laboratory scale formulation in aqueous solution.

#### 3.6.2. Process and LCI

Different NCs have been developed (Section 2.2) to encapsulate selected EOs, shielding them with good stability, controlled delivery, and improved efficacy. Primary data has been derived from the experimental work of this study during the production process (see Table 1), including energy flows modeled using the European average grid mix. Additionally, proxies from the Ecoinvent v3.11 database were used to complete the analysis where specific components or chemicals were not available. The Inventory data was set based on formulations for NC synthesis with zein protein (modified) and GZA-copolymer, normalized for the functional unit of 100 mL. Ecoinvent proxies are described in Table 5.

Several authors have researched a similar scope regarding the mechanism of NP formation, the most used raw materials, the physicochemical properties of EO-loaded NPs, and their application in several fields [42,43,44]. EOs can be extracted from various plant organs using different methods, and their environmental impact depends on several factors including the geographical origin of the plants, the extraction or distillation method used, the efficiency of the process, and energy consumption or recovery strategies. In this evaluation, due to the unavailability of specific Ecoinvent datasets, the calculations were merely based on average and estimated energy consumption average values for the extraction of each type of oil [43,44,45]. Resource utilization varies widely depending on the specific oil being produced; therefore, more detailed research is recommended to assess the entire EO value chain comprehensively.

All inventory data for the processes studied are based on experimental measurements at lab scale, including all inputs and outputs. For the LCA modeling, ecoinvent modules were selected for background processes, such as electricity supply or chemical production. In some cases, these modules act as proxies, while in others, they represent the best available datasets (e.g., for commonly produced chemicals).

#### 3.6.3. Results and Interpretation

##### Environmental Performance of the Emerging NCs

LCA calculations were performed using the Ecoinvent database version 3.11 and the Environmental Footprint 3.1 (adapted) impact assessment method, as recommended by the European Commission (Commission Recommendations 2013/179/EU) [21].

The results are presented in terms of environmental impact values (normalization and weighting set) obtained through the Characterization and the Single Score, measured in Eco-Points (Pts).

Zein-Based Core–Shell NCs Containing EOs

**Figure 17:**
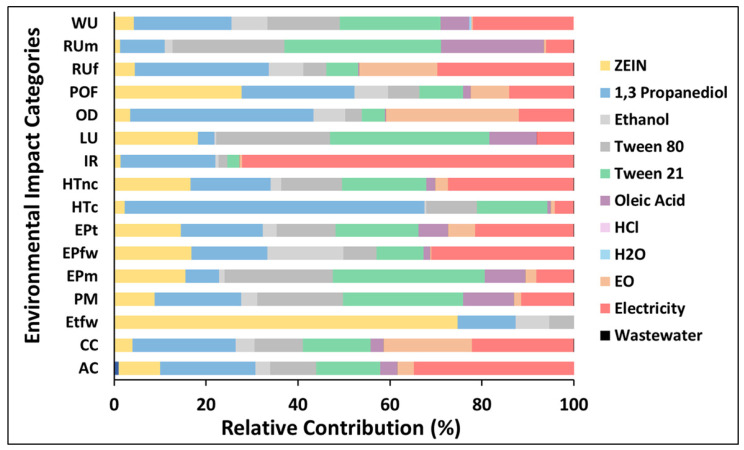
Zein-based NCs containing EOs (relative contributions). The following abbreviations are used: Acidification (AC); Climate change (CC); Ecotoxicity freshwater (Etfw); Particulate matter (PM); Eutrophication marine (EPm); Eutrophication freshwater (EPfw); Eutrophication terrestrial (EPt); Human toxicity cancer (HTc); Human toxicity non-cancer (HTnc); Ionizing radiation (IR); Land use (LU); Ozone depletion (OD); Photochemical ozone formation (POF); Resource use fossils (RUf); Resource use minerals and metals (RUm); Water use (WU).

Based on an example of NCs based on non-modified zein, Figure 17 highlights that the use of PDO and surfactants constitutes the largest contributor to the environmental impact categories. PDO, necessary for the production of NCs, overall represents a critical environmental issue; its contribution is estimated between 7% (“Euthrophication”) and 66% in “Ecotoxicity freshwater”. Therefore, it is possible to observe that all impact categories are affected by the consumption of PDO. However, the surfactants also determine a relevant impact contribution for most of the categories, impacting similarly 58% in “land use” and “resource use minerals and metals”. Zein production plays a key role in the impact of the categories “Ecotoxicity, freshwater” and “photochemical ozone”, and electricity consumption has a relative contribution between 4% and 72% across all categories. For the analysis of EO in the final product (i.e., NC), only electricity for its extraction process was inventoried, and that is why climate change and ozone depletion categories show higher contributions.

GZA-Based Core–Shell NCs Containing EOs

**Figure 18:**
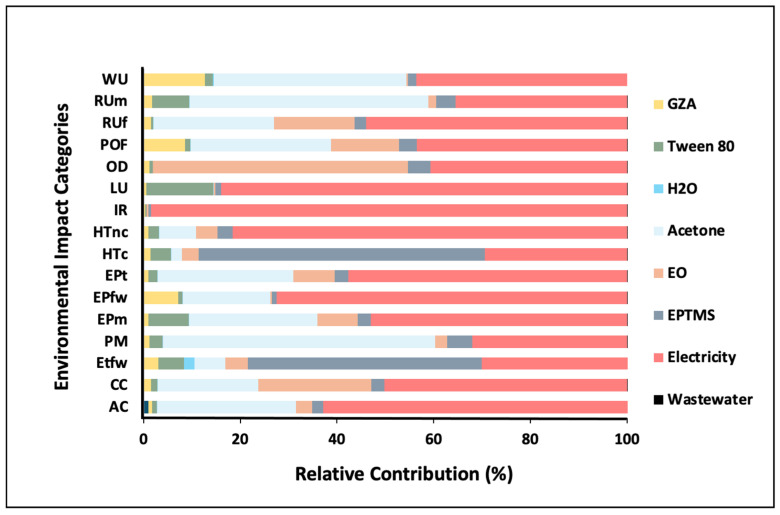
GZA-EPTMS-based NCs containing EOs (relative contributions).

GZA-EPTMS-based NCs have been selected for presentation of indicative results in this work. In contrast to zein-based NCs, the production of GZA-based NCs requires higher energy input, which is reflected in the relative impact category values shown in Figure 18.

These values indicate increased environmental impacts across all categories, ranging from 23% to 98%. Additionally, the use of EPTMS as a modifier and crosslinking agent for GZA contributed primarily to the “Ecotoxicity freshwater” and “Human toxicity” categories. It is also important to highlight that the use of acetone significantly contributed to several impact categories: 56% to “Particulate matter formation”, 49% to “Fossil resource use”, and 40% to the “Water use” category.

#### 3.6.4. Comparative LCA Results Focused on Shell Polymer Selection

Although the single-score LCA provides a useful summary of the overall environmental impact of a product or material, it should not be viewed as the sole criterion for decision-making. In the figure, the single score clearly indicates that a GZA-based NC has a higher total environmental impact (~35 μPt) compared to a zein NC (~25 μPt). This means that GZA-loaded NCs contribute more to environmental burdens across the measured categories.

**Figure 19:**
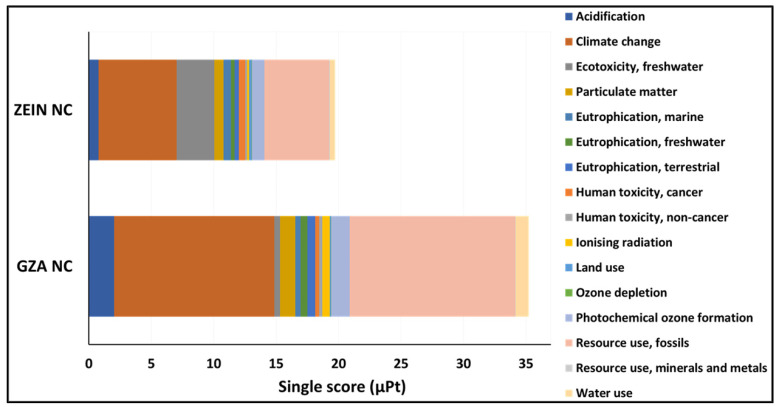
Comparative LCA of zein and GZA NCs: Single Score results.

The comparative analysis of the environmental impacts between zein-based NCs and GZA-based ones reveals that zein demonstrates a notably lower overall burden, making it the more sustainable option under the evaluated scenario (Figure 19). “Climate change” and “Fossil resource use” are the dominant contributors to the total impact in both materials, but these are significantly more pronounced in GZA-based NCs, which also exhibit higher values across several other categories such as “Water use”, “Human toxicity” (cancer and non-cancer), and “Photochemical ozone formation”.

The more compact distribution of impact categories in zein NCs suggests a reduced environmental footprint, supporting their suitability for applications where lower life cycle impacts are critical. However, other factors, such as material performance, cost, availability, and technical compatibility, must also be considered alongside the LCA results to ensure a balanced and context-specific selection in sustainable material decision-making.

#### 3.6.5. Comparative LCA Results Focused on Core Selection

**Figure 20:**
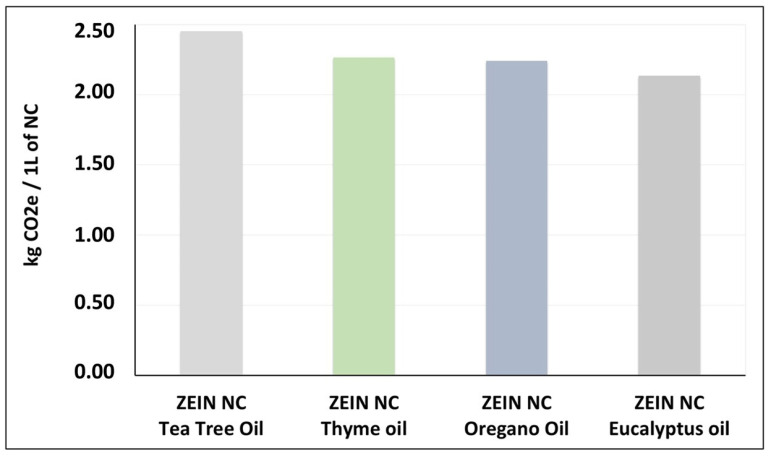
Impact of essential oil type on the carbon footprint of zein-based nanocapsules.

Figure 20 presents the comparative environmental impacts of four NC synthesis scenarios based on zein as the biopolymeric shell material, each involving a different EO as the core active ingredient, thereby illustrating the influence of core selection on the overall sustainability profile of the system. The assessment results for the EOs are derived from the energy demand associated with their extraction, specifically using conventional thermal distillation, which significantly influences two key impact categories: “Climate change” and “Resource use”.

These outcomes effectively represent the carbon footprint of NCs associated with each type of EO (core). The results stress that yield and energy consumption, which vary depending on the oil type, are critical parameters in determining the environmental performance of the NCs. Therefore, while the type of EO was found to significantly influence the overall environmental impact of the NCs, it was also identified in this study as a strategic factor in material selection, highlighting that sustainable NC synthesis depends not only on the choice of shell material but also on the energy-intensive nature of the core component, which directly affects the system’s environmental performance.

The estimated carbon footprint of these NC dispersions is approximately 2–2.5 kg CO_2_e per liter at laboratory scale.

Most energy values in this study were estimated by scaling from the eucalyptus distillation case reported by Lainez-Cerón et al. [45], which served as the primary reference. In their experiment, conventional hydro-distillation of eucalyptus using 6 g/min steam over 180 min (plant-to-water ratio 1:5) yielded ~1.19% EuO. Scaling this to 84 kg of fresh biomass required ~90.7 kg of steam (~199 MJ latent heat). Assuming 85–90% boiler efficiency, the net thermal energy demand was estimated at ~215 MJ per kg of EuO.

This value was used as a baseline to estimate energy demand for other herbs with similar yields (~1%), adjusted for biomass structure. Lavender and thyme, both aromatic herbs with semi-woody stems and moderate density, were assigned a value of 300 MJ/kg, representing typical thermal energy demand under conventional distillation [46]. Oregano, being less woody and with thinner leaves, was estimated at 285 MJ/kg, reflecting slightly faster steam penetration and shorter distillation time. These values align with industrial reports for peppermint oil, which can reach 330 MJ/kg under standard steam distillation [47].

For TTO, due to its fibrous and dense biomass and lower oil yield, a higher energy input of 425 MJ/kg was assumed, extrapolated from Lainez-Cerón et al. [45] and consistent with ranges reported for steam-distilled woody species.

Given the low batch volumes and process inefficiencies typical of lab-scale synthesis, the NCs’ carbon footprint is expected to decrease significantly upon upscaling to pilot or industrial production. Future work should explore how process scaling and renewable energy inputs could reduce environmental impact.

#### 3.6.6. Sensitivity Analysis on the Influence of Energy Use in LCA Results

In the experimental work conducted in the framework of this study, two types of GZA-based NCs—one with and one without crosslinking—were developed, whereas two modification temperatures, i.e., 30 °C and 80 °C, were investigated for non-crosslinked NCs. These systems have not been selected for detailed presentation and discussion in this manuscript, but they are good candidates for representing the sensitivity analysis related to energy consumption for similar NCs obtained using differences in the production steps.

**Figure 21:**
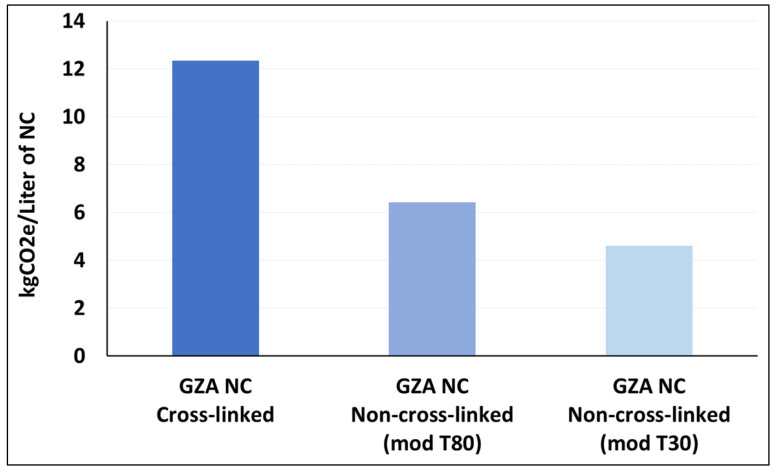
Carbon footprint evaluation of three scenarios with varying cross-linking conditions.

The current cradle-to-gate LCA evaluated the environmental impact, particularly highlighting significant differences in energy consumption between the different scenarios. The crosslinked variant is expected to be more energy-consuming but can enable covalent bonding of NCs to various substrates, like textile fibers, improving fixation, whereas a non-crosslinked shell modified at lower temperature (i.e., T = 30 °C) will have a smaller carbon footprint but without improvement in covalent bonding (Figure 21). Therefore, it is difficult to decide which production route could provide better sustainability. However, further studies including the use phase of final products may clarify which approach offers greater benefits in terms of environmental impact and performance in real applications.

This study provides a first comprehensive LCA of the evaluated nanocapsules, although some general limitations apply. Lab-scale experiments can lead to higher energy-related impacts per functional unit, which could be reduced at larger scale using renewable energy and optimized process integration. The use phase and end-of-life, including potential nanomaterial releases, were not considered and remain important areas for future research. Finally, the lack of published LCAs for nanocapsules means the results should be interpreted as absolute indicators of environmental performance, a common challenge in current nanomaterial LCA studies.

## 4. Conclusions

In this study, core–shell sub-micro-/nanocapsules (NCs) were successfully developed to encapsulate various essential oils (EOs) with the aim of enhancing antimicrobial (AM) efficacy while minimizing cytotoxicity. Two types of shell polymers—zein and GZA—were chemically modified to improve the physicochemical properties and stability of the NCs. Encapsulation efficiencies exceeding 98% were achieved across all EO types, with DDA-modified GZA-based NCs showing the highest values (encapsulation efficacy >99.4%). Dynamic light scattering (DLS) analysis demonstrated that chemical modification and crosslinking significantly improved particle size distribution (PSD) and ζ-potential (i.e., dispersion stability). Encapsulation markedly reduced the cytotoxicity of raw EOs toward mammalian cells, with cell viability increasing from ~20% to 60–80% at EO concentrations of 200–300 μg/mL. On the other hand, antibacterial tests against Gram (+) *Staphylococcus aureus* bacteria revealed that all tested EOs exhibited strong antibacterial activity (MIC = 2 µg/mL), while encapsulated formulations retained their activity (MIC = 20–30 µg/mL), indicating partial preservation of their bioactivity. Among all formulations, EPTMS-modified GZA-based NCs containing oregano (OO) or thyme oil (TO) exhibited the best balance between antibacterial performance and reduced toxicity (cell viability >80% at MIC concentration of 20 μg/mL). However, life cycle assessment (LCA) indicated that zein-based NCs are more environmentally friendly than GZA-based NCs due to lower energy consumption and reduced material impact.

Despite these encouraging results, further challenges remain in optimizing capsule size, release kinetics, and shell composition to simultaneously maximize antibacterial efficacy, minimize cytotoxicity, and reduce environmental impact. Future studies should therefore focus on deeper mechanistic understanding of EO release behavior and bacteria–capsule interactions, evaluation against a broader spectrum of clinically relevant microorganisms, assessment of long-term stability under application-relevant conditions, and the development of scalable and greener synthesis strategies. Although further research is required to optimize the synthesis of core-shell NCs, this approach provides a promising foundation for the development of EO-loaded NCs as eco-friendly, safe, and effective antibacterial agents for use in applications across various fields, including healthcare, hygiene, and surface disinfection.

## Figures and Tables

**Table 1:** Formulations for NCs based on (modified) zein and GZA-copolymer.

	Zein (Empty NCs)	Zein/ Zein-VA (EO-Loaded NCs)	GZA (Empty NCs)	GZA-EPTMS/ GZA-DDA * (EO-Loaded NCs)
**Shell-polymer, g**	3.00	3.00	2.00	2.00
**PDO, g**	16.00	16.00	-//-	-//-
**Ethanol, g**	7.00	7.00	-//-	-//-
**Tween 20, g**	5.00	5.00	-//-	-//-
**Tween 80, g**	7.0	7.0	1.25	1.25
**Oleic acid, g**	2.0	2.0	-//-	-//-
**HCl (1 M), g**	0.02	0.02	-//-	-//-
**EO, g**	-//-	2.0	-//-	5.00
**H_2_O, g**	59.98	57.98	50.00	50.00
**Acetone, g**	-//-	-//-	46.75	41.75
**Total, g**	**100**	**100**	**100**	**100**
**EPTMS, g**				3 *

*—Addition of EPTMS was only used for crosslinking of GZA-DDA-based NCs.

**Table 2:** Functional unit and key parameters introduced in SimaPro software.

**Functional unit**	100 mL
**Method**	EF3.1 (adapted)
**Geography**	Europe
**Time period**	2022–2023 lab data
**Data set**	Ecoinvent v3.11
**Data type**	Primary data (IVW experimental cases)
**Software**	SimaPro 10.2.0.1

**Table 4:** MIC and MBC values for raw and encapsulated EOs against Gram (+) *S. aureus* bacteria.

Material	MIC, µg/mL	MBC, µg/mL
Raw OO	2	5
Zein-VA NCs filled with OO	20	500
GZA-EPTMS NCs filled with OO	20	50
GZA-DDA NCs filled with OO	100	100
Raw TO	2	2
Zein-VA NCs filled with TO	500	500
GZA-EPTMS NCs filled with TO	20	200
GZA-DDA NCs filled with TO	20	200
Raw EuO	50	50
Zein-VA NCs filled with EuO	>500	>500
GZA-EPTMS NCs filled with EuO	30	200
GZA-DDA NCs filled with EuO	>500	>500
Raw TTO	5	50
Zein-VA NCs filled with TTO	>500	>500
GZA-EPTMS NCs filled with TTO	30	200
GZA-DDA NCs filled with TTO	>500	>500

**Table 5:** LCA proxies for NC simulation (FU: 100 mL, aqueous solution/production at laboratory scale).

Data	Material	Ecoinvent Proxy
**Product**	**Nanocapsule**	Created (100 mL)
**Inputs**	Shell: zein-based	Created (based on Maize at farm/DE mass, ethanol, water and electricity {RER})
	Shell: GZA-based	Created (based on dicarboxylic anhydride {RER}, ethanol {RER}, water deionized {RER}, and electricity {RER})
	Core: EO	Created (based on energy consumption for extraction process)
	Oleic Acid	Surfactant production, fatty acid derivate|Cut-off, U
	PDO	Propylene glycol, liquid {RER}|market for propylene glycol, liquid|Cut-off, included in the modelling as an available PDO proxy in Ecoinvent 3.11
	Water	Water, deionized {Europe without Switzerland}|market for water, deionized|Cut-off, U
	Ethanol	Ethanol, without water, in 99.7% solution state, from ethylene {RER}|market for ethanol, without water, in 99.7% solution state, from ethylene|Cut-off, U
	HCl	Hydrochloric acid (30% HCl) {RER}|
	Tween 20 Tween 80	Non-ionic surfactant {GLO}|non-ionic surfactant production, Fatty acid derivate|Cut-off, U
	EPTMS	Epoxy resin {RER}|epoxy resin production|Cut-off, U as an available proxy in Ecoinvent 3.11
	Electricity	Electricity, low voltage {DE}|market for|Cut-off, U
**Outputs**	Wastewater	Wastewater, average {Europe without Switzerland}|market for wastewater, average|Cut-off, U

**Table 3:** Concentration of ORO in S10 solution and encapsulation efficiency of EO via the synthesis of different NC-types.

Encapsulated EO	Shell Polymer	$\chi_{f}$ **, mol/L**	$E_{ef}$ **, %**
Oregano oil (OO)	VA-modified zein	7.34394 × 10^−6^	98.50
	EPTMS-modified GZA	1.22399 × 10^−5^	99.01
	DDA-modified GZA	6.11995 × 10^−6^	99.50
Thyme oil (TO)	VA-modified zein	7.61262 × 10^−6^	98.45
	EPTMS-modified GZA	1.16428 × 10^−5^	98.45
	DDA-modified GZA	5.55274 × 10^−6^	99.55
Eucalyptus oil (EuO)	VA-modified zein	7.14691 × 10^−6^	98.54
	EPTMS-modified GZA	1.25384 × 10^−5^	98.98
	DDA-modified GZA	6.44834 × 10^−6^	99.47
Tea tree oil (TTO)	VA-modified zein	6.44834 × 10^−6^	98.68
	EPTMS-modified GZA	1.30758 × 10^−5^	98.93
	DDA-modified GZA	7.16482 × 10^−6^	99.41

## Data Availability

The data presented in this study are available upon request from the corresponding author.

## References

[1] Spernovasilis N., Tsiodras S., Poulakou G. (2022). Emerging and Re-Emerging Infectious Diseases: Humankind’s Companions and Competitors. Microorganisms.

[2] Morens D.M., Fauci A.S. (2013). Emerging Infectious Diseases: Threats to Human Health and Global Stability. PLoS Pathog..

[3] Majorin F., Freeman M.C., Barnard S., Routray P., Boisson S., Clasen T. (2014). Child Feces Disposal Practices in Rural Orissa: A Cross Sectional Study. PLoS ONE.

[4] The Global Handwashing Partnership. https://globalhandwashing.org/about-handwashing/why-handwashing/health/.

[5] Kaloustian J., Chevalier J., Mikail C., Martino M., Abou L., Vergnes M.F. (2008). Étude de six huiles essentielles: Composition chimique et activité antibactérienne. Phytothérapie.

[6] Benjilali B., Ayadi A. (1986). Methode d’études des propriétes antiseptiques des huiles essentielles par contact direct en milieu gelose [Thymus capitatus, Rosmarinus officinalis, Eucalyptus globulus, Artemisia herba alba]. Plantes Méd. Phytothér..

[7] Burt S. (2004). Essential oils: Their antibacterial properties and potential applications in foods—A review. Int. J. Food Microbiol..

[8] Stefanakis M.K., Touloupakis E., Anastasopoulos E., Ghanotakis D., Katerinopoulos H.E., Makridis P. (2013). Antibacterial activity of essential oils from plants of the genus Origanum. Food Control.

[9] Pontes-Quero G.M., Esteban-Rubio S., Pérez Cano J., Aguilar M.R., Vázquez-Lasa B. (2021). Oregano essential oil micro-and nanoencapsulation with bioactive properties for biotechnological and biomedical applications. Front. Bioeng. Biotechnol..

[10] Jobdeedamrong A., Jenjob R., Crespy D. (2018). Encapsulation and release of essential oils in functional silica nanocontainers. Langmuir.

[11] Duncan B., Li X., Landis R.F., Kim S.T., Gupta A., Wang L.-S., Ramanathan R., Tang R., Boerth J.A., Rotello V.M. (2015). Nanoparticle-Stabilized Capsules for the Treatment of Bacterial Biofilms. ACS Nano.

[12] Zhu Z., Min T., Zhang X., Wen Y. (2019). Microencapsulation of thymol in poly(lactide-co-glycolide) (PLGA): Physical and antibacterial properties. Materials.

[13] Yoncheva K., Benbassat N., Zaharieva M.M., Dimitrova L., Kroumov A., Spassova I., Kovacheva D., Najdenski H.M. (2021). Improvement of the Antimicrobial Activity of Oregano Oil by Encapsulation in Chitosan—Alginate Nanoparticles. Molecules.

[14] Majidi Yazdi A., Hosseini A., Gheibihayat S.M., Beygi M., Haghiralsadat B.F., Oroojalian F. (2024). Characterization, cell toxicity, and antimicrobial activity of a carvacrol-encapsulating nanoliposomal system against *Staphylococcus aureus*, *Pseudomonas aeruginosa*, and *Escherichia coli*. Nanomed. J..

[15] Cimino C., Maurel O.M., Musumeci T., Bonaccorso A., Drago F., Souto E.M.B., Pignatello R., Carbone C. (2021). Essential oils: Pharmaceutical applications and encapsulation strategies into lipid-based delivery systems. Pharmaceutics.

[16] Buday-Malik A., Velősy A., Józsa Z., Mannheim V., Terjék A., Fekete L., Szita K.T. The Role of LCA in Innovative Product Development. Proceedings of the 27th International Sustainable Development Research Society Conference.

[17] Nizam N.U.M., Hanafiah M.M., Woon K.S. (2021). A Content Review of Life Cycle Assessment of Nanomaterials: Current Practices, Challenges, and Future Prospects. Nanomaterials.

[18] DocCheck. https://flexikon.doccheck.com/de/Oil_Red_O.

[19] (2012). Methods for Dilution Antimicrobial Susceptibility Tests for Bacteria that Grow Aerobically, Approved Standard M07-A9, 9th ed..

[20] (1998). Methods for Determining Bactericidal Activity of Antimicrobial Agents. Approved Guideline M26-A.

[21] (2006). Environmental Management—Life Cycle Assessment—Requirements and Guidelines.

[22] LibreTexts Chemistry Spectroscopy of Carboxylic Acid Derivatives: IR Characterization of Anhydrides and Related Compounds. https://chem.libretexts.org/Bookshelves/Organic_Chemistry/Organic_Chemistry_%28Morsch_et_al.%29/21%3A_Carboxylic_Acid_Derivatives-_Nucleophilic_Acyl_Substitution_Reactions/21.10%3A_Spectroscopy_of_Carboxylic_Acid_Derivatives.

[23] Corradini E., Curti P.S., Meniqueti A.B., Martins A.F., Rubira A.F., Muniz E.C. (2014). Recent advances in food-packing, pharmaceutical and biomedical applications of zein and zein-based materials. Int. J. Mol. Sci..

[24] Khan R., Rashid S., Khan S., Almutawif Y.A., Pari B. (2024). Synthesis and evaluation of vanillin Schiff bases as potential antimicrobial agents against ESBL-producing bacteria: Towards novel interventions in antimicrobial stewardship. Sci. Rep..

[25] Jung D.-M., de Ropp J.S., Ebeler S.E. (2000). Study of Interactions between Food Phenolics and Aromatic Flavors Using One- and Two-Dimensional 1H NMR Spectroscopy. J. Agric. Food Chem..

[26] Fijałkowska A., Wesołowska A., Rakoczy R., Jedrzejczak-Silicka M. (2024). A comparative study of thyme (*Thymus vulgaris* L.) essential oils and thymol–differences in chemical composition and cytotoxicity. Chem. Process Eng. New Front..

[27] García-Salinas S., Elizondo-Castillo H., Arruebo M., Mendoza G., Irusta S. (2018). Evaluation of the antimicrobial activity and cytotoxicity of different components of natural origin present in essential oils. Molecules.

[28] Jamil B., Abbasi R., Abbasi S., Imran M., Khan S.U., Ihsan A., Javed S., Bokhari H., Imran M. (2016). Encapsulation of cardamom essential oil in chitosan nano-composites: In-vitro efficacy on antibiotic-resistant bacterial pathogens and cytotoxicity studies. Front. Microbiol..

[29] Beyaz H., Kavaz D., Rizaner N. (2025). Chitosan nanoparticle encapsulation of thymus capitatus essential oil: In vitro release, antioxidant, antibacterial activity and cytotoxicity in MDA-MB-231 cells. Pharm. Dev. Technol..

[30] Wadhwa G., Kumar S., Mittal V., Rao R. (2019). Encapsulation of babchi essential oil into microsponges: Physicochemical properties, cytotoxic evaluation and anti-microbial activity. J. Food Drug Anal..

[31] Chiriac A.P., Rusu A.G., Nita L.E., Chiriac V.M., Neamtu I., Sandu A. (2021). Polymeric Carriers Designed for Encapsulation of Essential Oils with Biological Activity. Pharmaceutics.

[32] Carbone C., Martins-Gomes C., Caddeo C., Silva A.M., Musumeci T., Pignatello R., Puglisi G., Souto E.B. (2018). Mediterranean essential oils as precious matrix components and active ingredients of lipid nanoparticles. Int. J. Pharm..

[33] Dorman H.J.D., Deans S.G. (2000). Antimicrobial agents from plants: Antibacterial activity of plant volatile oils. J. Appl. Microbiol..

[34] Lambert R.J.W., Skandamis P.N., Coote P.J., Nychas G.J.E. (2001). A study of the minimum inhibitory concentration and mode of action of oregano essential oil, thymol and carvacrol. J. Appl. Microbiol..

[35] Soković M., Glamočlija J., Marin P.D., Brkić D., Van Griensven L.J. (2010). Antibacterial effects of the essential oils of commonly consumed medicinal herbs using an in vitro model. Molecules.

[36] Raman A., Weir U., Bloomfield S.F. (1995). Antimicrobial effects of tea-tree oil and its major components on *Staphylococcus aureus*, Staph. epidermidis and Propionibacterium acnes. Lett. Appl. Microbiol..

[37] Carson C.F., Hammer K.A., Riley T.V. (2006). Melaleuca alternifolia (tea tree) oil: A review of antimicrobial and other medicinal properties. Clin. Microbiol. Rev..

[38] Mulyaningsih S., Sporer F., Zimmermann S., Reichling J., Wink M. (2010). Synergistic properties of the terpenoids aromadendrene and 1, 8-cineole from the essential oil of Eucalyptus globulus against antibiotic-susceptible and antibiotic-resistant pathogens. Phytomedicine.

[39] Abdulsalam R.A., Ijabadeniyi O.A., Sabiu S. (2024). Fatty acid-modified chitosan and nanoencapsulation of essential oils: A snapshot of applications. Carbohydr. Res..

[40] Weisany W., Yousefi S., Soufiani S.P., Pashang D., McClements D.J., Ghasemlou M. (2024). Mesoporous silica nanoparticles: A versatile platform for encapsulation and delivery of essential oils for food applications. Adv. Colloid Interface Sci..

[41] Gonçalves N.D., de Lima Pena F., Sartoratto A., Derlamelina C., Duarte M.C.T., Antunes A.E.C., Prata A.S. (2017). Encapsulated thyme (*Thymus vulgaris*) essential oil used as a natural preservative in bakery product. Food Res. Int..

[42] (2023). Updated Characterisation and Normalisation Factors for the Environmental Footprint 3.1 Method.

[43] Lammari N., Louaer O., Meniai A.H., Elaissari A. (2020). Encapsulation of Essential Oils via Nanoprecipitation Process: Overview, Progress, Challenges and Prospects. Pharmaceutics.

[44] Carvalho A., Mimoso A.F., Mendes A.N., Matos H. (2014). From a literature review to a framework for environmental process impact assessment index. J. Clean. Prod..

[45] Lainez-Cerón E., Jiménez-Munguía M.T., López-Malo A., Ramírez-Corona N. (2021). Effect of process variables on heating profiles and extraction mechanisms during hydrodistillation of eucalyptus essential oil. Heliyon.

[46] Gavahian M., Chu Y.H. (2018). Ohmic accelerated steam distillation of essential oil from lavender in comparison with conventional steam distillation. Innov. Food Sci. Emerg. Technol..

[47] Kant R., Kumar A. (2023). Thermo-enviro-economic analysis of conventional steam distillation system for peppermint oil extraction. Therm. Sci. Eng. Prog..

